# A Quinoxaline Derivative as a Potent Chemotherapeutic Agent, Alone or in Combination with Benznidazole, against *Trypanosoma cruzi*

**DOI:** 10.1371/journal.pone.0085706

**Published:** 2014-01-17

**Authors:** Jean Henrique da Silva Rodrigues, Tânia Ueda-Nakamura, Arlene Gonçalves Corrêa, Diego Pereira Sangi, Celso Vataru Nakamura

**Affiliations:** 1 Programa de Pós-Graduação em Ciências Biológicas – Biologia Celular e Molecular, Universidade Estadual de Maringá, Maringá, Paraná, Brazil; 2 Departamento de Ciências Básicas da Saúde - Laboratório de Inovação Tecnológica no Desenvolvimento de Fármacos e Cosméticos, Universidade Estadual de Maringá, Maringá, Paraná, Brazil; 3 Departamento de Química - Laboratório de Síntese de Produtos Naturais, Universidade Federal de São Carlos, São Carlos, São Paulo, Brazil; Instituto Butantan, Laboratório Especial de Toxinologia Aplicada, Brazil

## Abstract

**Background:**

Chagas’ disease is a condition caused by the protozoan *Trypanosoma cruzi* that affects millions of people, mainly in Latin America where it is considered endemic. The chemotherapy for Chagas disease remains a problem; the standard treatment currently relies on a single drug, benznidazole, which unfortunately induces several side effects and it is not successful in the cure of most of the chronic patients. In order to improve the drug armamentarium against Chagas’ disease, in the present study we describe the synthesis of the compound 3-chloro-7-methoxy-2-(methylsulfonyl) quinoxaline (quinoxaline **4**) and its activity, alone or in combination with benznidazole, against *Trypanosoma cruzi in vitro*.

**Methodology/Principal Findings:**

Quinoxaline **4** was found to be strongly active against *Trypanosoma cruzi* Y strain and more effective against the proliferative forms. The cytotoxicity against LLCMK_2_ cells provided selective indices above one for all of the parasite forms. The drug induced very low hemolysis, but its anti-protozoan activity was partially inhibited when mouse blood was added in the experiment against trypomastigotes, an effect that was specifically related to blood cells. A synergistic effect between quinoxaline **4** and benznidazole was observed against epimastigotes and trypomastigotes, accompanied by an antagonistic interaction against LLCMK_2_ cells. Quinoxaline 4 induced several ultrastructural alterations, including formations of vesicular bodies, profiles of reticulum endoplasmic surrounding organelles and disorganization of Golgi complex. These alterations were also companied by cell volume reduction and maintenance of cell membrane integrity of treated-parasites.

**Conclusion/Significance:**

Our results demonstrated that quinoxaline **4**, alone or in combination with benznidazole, has promising effects against all the main forms of *T. cruzi*. The compound at low concentrations induced several ultrastructural alterations and led the parasite to an autophagic-like cell death. Taken together these results may support the further development of a combination therapy as an alternative more effective in Chagas’ disease treatment.

## Introduction

The American trypanosomiasis, also known as Chagas’ disease, is a major public health problem that affects more than 10 million people worldwide, mostly in Latin America where it is considered endemic. [1] The causative agent of Chagas’ disease is the hemoflagellate protozoan *Trypanosoma cruzi* that belongs to Trypanosomatidae family. This taxon also includes other parasites that are responsible for relevant diseases, such as *Trypanosoma brucei* (Human African Trypanosomiasis) and *Leishmania spp.* (leishmaniasis). With regard to its clinical aspects, Chagas’ disease has a variable presentation and progression. [2] Most infected patients will never develop any symptoms, characterizing the indeterminate form of chronic Chagas’ disease. Approximately 30% of infected individuals will develop severe digestive or cardiac symptoms, [3] making this infection the most common cause of myocarditis worldwide. [4].

The available treatment for Chagas’ disease is restricted to only two nitroheterocyclic compounds, benznidazole and nifurtmox, that have been used to treat Chagas’ disease for more than 40 years. [5] Although both drugs are trypanocidal against all three forms of the parasite, their high systemic toxicity is important to consider, which can be expressed in a variety of symptoms, such as anorexia, nausea, headache, psychological disorders, polyneuropathies, and dermatitis. [6] Their efficacy is still considered low and restricted to acute manifestations of the disease. These drugs successfully cure approximately 20% and 80% of chronic and acute Chagas’ disease patients, respectively. [7] Thus, new drugs and therapies should be sought for the treatment of Chagas’ disease.

Several studies have been conducted to find new active compounds against *T. cruzi*. The search based on natural products is promising, with the possibility of screening a high number of plants and isolate active compounds with low toxicity. [8]–[9] Another approach that shows potential is the design and evaluation of synthetic compounds, either alone or in combination, against the parasite. [10]–[12] Among the groups of synthetic molecules, quinoxalines are a class of heterocyclic compounds that have been intensively studied for their biological activities, showing promising effect against bacteria, fungi, [13] tumors, [14] viruses, [15] and protozoa. [16]–[17] A library of new quinoxaline derivatives was recently tested against *Leishmania peruviana* and *T. cruzi* and showed interesting results. [18].

Combination therapy is an important way to improve the effectiveness of available drugs with different mechanisms of action in the treatment of several disorders, especially in the control of infectious diseases. [19] It is an approach that is currently used for the treatment of cancer, [20] osteoarthits, [21] tuberculosis, [22] acquired immune deficiency syndrome, [23] and opportunistic infections. [24] Compared with monotherapy, combination therapy presents higher cure rates, reduced treatment times, lower side effects, and delays in the selection of resistant parasites. [19].

Combination therapies based on the available drugs for Chagas’ disease treatment, benznidazole and nifurtimox, and novel uses of old drugs, such as allopurinol, itraconazole, ketoconazole, and fluconazole, have been proposed as a promising therapeutic alternative for patients with acute and chronic Chagas’ disease. [7], [25].

Considering that no effective vaccine against Chagas’ disease exists and the controversial and inefficient chemotherapy available to patients, new therapies are required. [5] Thus, the present study investigated the anti-protozoan activity and the main alterations induced by a quinoxaline derivative, 3-chloro-6-methoxy-2-(methylsulfonyl)quinoxaline (**4**), against the three main forms of *T. cruzi* and assessed the safety of this compound by testing its toxicity against cultured mammalian cells and human red blood cells. We also verified the *in vitro* activity of quinoxaline **4** and benznidazole combined against *T. cruzi* and mammalian cells to determine whether this combination has potential as a future multi-drug therapy.

## Methods

### Synthesis of 3-Chloro-7-methoxy-2-(methylsulfonyl)quinoxaline (4)

Unless otherwise noted, all of the commercially available reagents were purchased from Aldrich Chemical Co. and used without purification. ^1^H and ^13^C nuclear magnetic resonance (NMR) spectra were recorded with a Bruker ARX-400 (400 and 100 MHz, respectively). The infrared (IR) spectra refer to tablets in KBr and were measured with a Bomem M102 spectrometer. Mass spectra were recorded with a Shimadzu GCMS-QP5000. Analytical thin-layer chromatography was performed on a 0.25 µm film of silica gel that contained the fluorescent indicator UV_254_ supported on an aluminum sheet (Sigma-Aldrich). Flash column chromatography was performed using silica gel (Kieselgel 60, 230–400 mesh, E. Merck). Gas chromatography was performed using a Shimadzu GC-17A with N_2_ as the carrier and a DB-5 column. Melting points were performed in Microquimica MQAPF-301.

### 3-Methoxy-*N*-[1-(methylthio)-2-nitroethenyl]-benzenamine (2) [26]

Nitroethene **1** (0.182 mmol), *m*-methoxyaniline (0.182 mmol), and ethanol (1 mL) were placed in a glass tube, purged with oxygen-free nitrogen for 10 min, sealed, and irradiated for 90 min in a CEM Discovery focused microwave oven at 110°C and 70 W. The crude product was purified by flash chromatography using hexane:ethyl acetate (4∶1 ratio) as the eluent, furnishing nitroketene *N,S*-acetal **2** in 91% yield (melting point: 125–126°C). ^1^H NMR (400 MHz, CDCl_3_) δ: 2.39 (s, 3H), 3.83 (s, 3H), 6.70 (s, 1H), 6.90 (t, 1H, *J* 2.15 Hz), 6.92–6.87 (m, 2H), 7.33 (t, 1H, *J* 8.26 Hz), 11.82 (s, 1H). ^13^C NMR (100 MHz, CDCl_3_) δ: 14.71, 54.01, 111.44, 113.76, 117.89, 130.09, 137.19, 160.25, 163.28. IR (KBr) ν_max_/cm^−1^: 3159.17, 3070.45, 2995.23, 1602.73, 1546.80, 1465.79, 1417.58, 1330.79, 1269.07, 1222.78, 1157.20, 958.55, 848.62, 684.68, 570.89, 532.31. GC-MS (70 eV) *m/z* (%): 240 (M^+^, 12), 206 (41), 159 (38), 147 (64), 107 (100), 77 (99).

### 2-Chloro-7-methoxy-3-methylsulfanylquinoxaline (3) [27]

To a suspension of nitroketene *N*,*S*-acetal **2** (0.208 mmol) in acetonitrile (1 mL), POCl_3_ (0.625 mmol) was added dropwise at 0°C for 15 min. The mixture was stirred at 80°C for 4 h and cooled to room temperature, and a saturated solution of NaHCO_3_ (2.5 mL) was added. The aqueous solution was extracted with CHCl_3_ (3×2 mL). The combined organic phases were washed with water (2×2 mL) and brine (2 mL) and dried over anhydrous Na_2_SO_4_. The solvent was removed under vacuum, and the crude product was purified with column chromatography using silica gel and hexane:ethyl acetate (9∶1) as the eluent, affording compound **3** in 54% yield (melting point: 109–111°C. ^1^H NMR (400 MHz, CDCl_3_) δ: 7.83–7.80 (m, 1H), 7.28–7.26 (m, 2H), 3.96 (s, 3H), 2.67 (s, 3H). ^13^C NMR (100 MHz, CDCl_3_) δ: 161.08, 157.10, 142.94, 134.68, 129.08, 121.18, 105.95, 99.99, 55.80, 13.76. IR (ν_max,_ KBr): 3006, 1616, 1494, 1213 cm^−1^. GC-MS (70 eV) *m/z*: 240 (M^+^, 100), 205 (88), 190 (35), 159 (40), 63 (29).

### 3-Chloro-7-methoxy-2-(methylsulfonyl)quinoxaline (4) [27]

To a solution of *m*-chloroperbenzoic acid (0.25 mmol) in dichloromethane (1 mL) was added a solution of quinoxaline **3** (0.11 mmol) in dichloromethane (1 mL) at 0°C for 2 h. Ice water (2 mL) was then added, and the organic layer was washed with a 10% aqueous solution of NaHCO_3_ (2 mL), water (2 mL), and brine (2 mL). It was dried over anhydrous Na_2_SO_4_ and concentrated under vacuum. The crude product was purified with column chromatography in silica gel and hexane:ethyl acetate (3∶2) as the eluent, affording quinoxaline **4** in 87% yield. ^1^H NMR (400 MHz, CDCl_3_) δ: 7.98 (d, *J = *9.26 Hz, 1H), 7.59 (dd, *J = *9.26; 2.85 Hz, 1H), 7.40 (d, *J = *2.85 Hz, 1H), 4.01 (s, 3H), 3.53 (s, 3H). ^13^C NMR (100 MHz, CDCl_3_) δ: 162.19, 150.00, 140.33, 139.07, 138.67, 129.23, 127.39, 106.68, 56.17, 40.27. CG-MS (70 eV) *m/z*: 272 (M^+^, 63), 210 (68), 193 (90), 158 (100), 117 (50), 77 (36).

Stock solutions of quinoxaline **4** were prepared aseptically in dimethylsulfoxide (DMSO; Sigma, St. Louis, MO, USA) at the time of use, with the final concentration of the experiments not exceeding 0.5%. All of the negative controls received the same concentration of DMSO as the treatment.

### Parasites and Cell Cultures

All of the experiments were performed with the Y strain of *Trypanosoma cruzi*. Epimastigote forms were cultivated in Liver Infusion Tryptose (LIT) medium [28] supplemented with 10% heat-inactivated fetal bovine serum (FBS; Gibco Invitrogen, Grand Island, NY, USA), kept at 28°C, and maintained by weekly transfers. Trypomastigotes were collected from the supernatant of LLCMK_2_ cells (*Macaca mulatta* epithelial kidney cells) preinfected by bloodstream trypomastigotes aseptically obtained by heart puncture of infected Swiss mice at the peak of parasitaemia. LLCMK_2_ cells, infected or not, were maintained in Dulbecco’s modified Eagle medium (DMEM; Gibco Invitrogen), pH 7.4, supplemented with 2 mM L-glutamine, 10% FBS, and 50 mg/L gentamicin at 37°C in a humidified 5% CO_2_ atmosphere. [29] Amastigotes and trypomastigotes present on the supernatant of infected LLCMK_2_ cells were separated by differential centrifugation at 850×*g* for 5 min. The elongated forms were harvested from the supernatant, and the round forms were collected from the pellet.

### Anti-proliferative Activity against Epimastigote Forms

Epimastigotes (1×10^6^ parasites/mL) in the exponential phase of growth (96 h) were harvested and incubated in the presence of LIT supplemented with 10% FBS added or not to increasing concentrations of quinoxaline **4** (0.37–37.0 µM). Parasites were incubated at 28°C in 24-well flat-bottom plates, collected aseptically every 24 h, and counted in a Neubauer hemocytometer. Benznidazole (Laboratório Central de Medicamentos, Pernambuco, Brazil) was used as the reference drug and counted after 96 h. The IC_50_ (concentration that inhibited 50% of parasite growth) and IC_90_ (concentration that inhibited 90% of parasite growth) were determined by regression analysis of the data.

### Activity against Trypomastigote Forms

Trypomastigotes, obtained from the supernatant of infected LLCMK_2_ cells, were inoculated (1×10^7^ parasites/mL) in DMEM and added to 96-well plates in the presence and absence of increasing concentrations of quinoxaline **4** (1.8–360.0 µM). Parasites were incubated for 24 h at 37°C in a 5% CO_2_ atmosphere. After incubation, the viability of the parasites was examined by mobility under a light microscope (Olympus CX31) using the Pizzi-Brener method. [30] The same experiment was performed three times with supplementation with 20% FBS, Swiss mouse blood, and Swiss mouse plasma. The concentrations that inhibited 50% (EC_50_) and 90% (EC_90_) of parasite viability were determined by regression analysis of the data.

### Activity against Intracellular Amastigotes

To assess the activity of quinoxaline **4** against the intracellular form of the parasite, the assay was carried out as described previously [31] with slight modifications. For that, LLCMK_2_ cells were harvested, resuspended in DMEM plus 10% FBS and plated (2.5×10^5^ cells/mL) in 24-well plates that contained round glass coverslips. When confluent growth was achieved, the cells were infected with trypomastigotes obtained from pre-infected cultures at a ratio of 10 parasites per 1 mammalian cell. After 24 h, the medium that contained the parasites was removed, the cells were washed in phosphate-buffered saline (PBS), and DMEM with or without increasing concentrations of quinoxaline **4** (1.8–18.0 µM) was added. The cells were maintained for 96 h at 37°C in a 5% CO_2_ atmosphere. After the incubation period, the glass coverslips were subjected to fixation with methanol and Giemsa staining and permanently prepared with Entellan (Merck, Germany). The number of infected cells and amastigotes was determined under a light microscope by counting randomly 200 cells in duplicate cultures. The results are expressed as the survival index (%). The survival index was obtained by multiplying the percentage of infected cells by the number of amastigotes per infected LLCMK_2_ cell. The survival index observed in the control without treatment was considered as 100%, the results for treated groups were comparatively evaluated.

### Cytotoxicity Assay

To evaluate the cytotoxicity of the quinoxaline derivate, the MTT assay was used. This colorimetric assay is based on the ability of viable mitochondria to convert MTT, a water-soluble tetrazolium salt (3-[4,5-dimethylthiazol-2-yl]-2,5-diphenyltetrazolium bromide), into an insoluble purple-colored formazan precipitate. [32] LLCMK_2_ cells were collected from confluent cultures, plated in 96-well plates, and incubated at 37°C in a humid 5% CO_2_ atmosphere. After 24 h, the medium was replaced with new DMEM that contained concentrations of quinoxaline **4** that ranged from 3.7 to 73.6 µM. Following 96 h incubation, the cells were washed in PBS, and 50 µL of MTT (2 mg/mL) was added to each well. The formazan crystals were solubilized in DMSO, and absorbance was read at 570 nm in a microplate reader (Bio Tek – Power Wave XS). The concentration that diminished 50% of the absorbance value observed in the control represented the CC_50_ (cytotoxic concentration for 50% of the cells).

### Hemolytic Activity

Another way to assess the safety of a drug is to measure its hemolytic activity. Blood (A+ type) from a healthy human donor was collected, defibrinated, and washed in glycosylated saline to remove any free hemoglobin from the defibrinization process. Red blood cells were inoculated in 96-well plates at 3% in glycosylated saline with different concentrations of quinoxaline **4** (3.7–1000 µM). The plates were incubated for 3 h at 37°C, and the supernatant was read at 550 nm. To calculate the percentage of hemolysis, 1% Triton X-100 was used as a positive control.

### Drug Combination Assay

To verify the effect of the combination of quinoxaline **4** and beznidazole on epimastigotes, trypomastigotes, and LLCMK_2_ cells, we applied the Combination Index method proposed by Chou and Talalay [33] and reviewed by Zhao. [34] The experimental design consists of combinations of at least four concentrations of each drug arranged in a checkerboard at a 1∶2 concentration ratio. Briefly, 1×10^6^ epimastigotes/mL were resuspended in LIT in the presence of different concentrations of both drugs and counted after 96 h incubation at 28°C. For the trypomastigote inhibition assay, cells were collected (1×10^7^ parasites/mL) and exposed to different concentrations of each drug in combination. After 24 h incubation, the parasites were analyzed for viability. In the cytotoxicity experiment, LLCMK_2_ cells were plated as described and exposed to different concentrations of both drugs. After 96 h, cell viability was quantified using the MTT method. The data were calculated and mathematically expressed as the Combination Index (CI = [IC_50benzo combined_/IC_50benzo_ _alone_]+[IC_50quinoxaline **4** combined_/IC_50quinoxaline **4**_ _alone_]; the numerators are the concentrations of each drug that in combination are active against 50% of the cells, and the denominators are the concentrations that have this same effect for each drug alone). The interpretation of the CI was based on the broadly used specifications established by Chou and Talalay, [33], [35] in which CI values less than, equal to, and more than 1 indicate synergism, additivity, and antagonism, respectively. The data were also graphically expressed as isobolograms.

### Electron Microscopy

To evaluate the effect of the compound on the morphology of the parasite by SEM, epimastigotes (1×10^6^ parasites/mL) and trypomastigotes (1×10^7^ parasites/mL) were treated as described previously with the compound at the concentrations that corresponded to the IC_50_ and IC_90_. After incubation, they were harvested, washed twice in PBS, and fixed with 2.5% glutaraldehyde in 0.1 M sodium cacodylate buffer at 4°C for 24 h. The parasites were then placed on a glass support covered with poly-L-lysine, dehydrated in crescent grades of ethanol, dried by the critical-point method with CO_2_, coated with gold, and observed on a Shimadzu SS-550 SEM. For the ultrastructure analysis, epimastigotes fixed as described above were postfixed in a solution of 1% OsO_4_, 0.8% potassium ferrocyanide, and 10 mM CaCl_2_ in 0.1 M cacodylate buffer. The samples were then dehydrated in an increasing acetone gradient and embedded in Polybed 812 resin. Ultrathin sections were then obtained, stained with uranyl acetate and lead citrate, and observed on a JEOL JM 1400 TEM. The parasites were analyzed and compared with controls without any treatment.

### Cell Volume Determination

Epimastigotes (1×10^6^ parasites/mL) treated for 24 h with different concentrations of quinoxaline **4** (1.8, 3.7, and 7.4 µM) were collected by centrifugation, washed twice in PBS, resuspended in PBS, and directly analyzed by fluorescence-activated cell sorting using a BD FACSCalibur flow cytometer (Becton-Dickinson, Rutherford, NJ, USA). A total of 10,000 events were acquired in a region previously established for the parasites. Parasites treated with actinomycin D (20.0 mM) were used as a positive control. Histograms were generated and the analysis was performed using CellQuest software (Joseph Trotter, The Scripps Research Institute, La Jolla, CA, USA), considering the FSC parameter that represents the cell volume.

### Cell Membrane Integrity Assay

Epimastigotes (1×10^6^ parasites/mL), trypomastigotes (1×10^7^ parasites/mL), and amastigotes (1×10^7^ parasites/mL) were incubated in the presence or absence of different concentrations of quinoxaline **4** (1.8, 3.7, and 7.4 µM) for 24 h. After incubation, the cells were harvested, washed twice in PBS, and marked with 0.2 µg/mL propidium iodide for 10 min. Digitonin (40.0 µM) was used as a positive control for the loss of cell membrane integrity. Data acquisition and analysis were performed using a FACSCalibur flow cytometer equipped with CellQuest software. A total of 10,000 events were acquired in the region previously established as the one that corresponded to the parasites.

### Statistical Analysis

All of the quantitative experiments were conducted in at least three independent experiments in duplicate. The statistical analyses were performed using GraphPad Prism 5.0 software. The data were analyzed using one-way analysis of variance (ANOVA), and the Tukey *post hoc* test was used to compare means when appropriate. Values of *p*<0.05 were considered statistically significant.

### Ethics Statement

For the hemolytic assay, the blood was obtained from healthy volunteer donors according to Declaration of Helsinki (Ethical principles for medical research involving human subjects) last reviewed in 2008. The donors received an explanation about the purpose of the study and provided the written consent before the blood collection. The blood was collected by brachial vein puncture by a trained professional with appropriate material and medical support. All procedures were conducted as described in specific protocol approved by the “Comitê de Ética em Pesquisa com Seres Humanos of the Universidade Estadual de Maringá” (acceptance 293/2006 COPEP-UEM). For the assays that involved mouse blood, Swiss and BALB/c mice were obtained from the Central Animal Facility of the Universidade Estadual de Maringá. All procedures were carried out in accordance with the guidelines established by the Committee on Ethics of Animal Experiments of the Universidade Estadual de Maringá, as stated in the detailed protocol approved for this experiment (acceptance 058/2010).

## Results

### Synthesis of 3-chloro-7-methoxy-2-(methylsulfonyl)quinoxaline (4)

**Figure 1:**
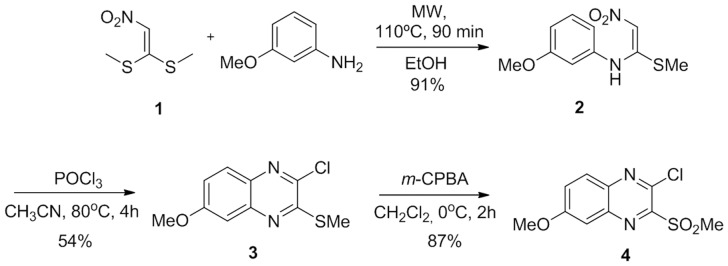
General procedure for the synthesis of 3-chloro-7-methoxy-2-(methylsulfonyl)quinoxaline (4).

The synthesis of quinoxaline **4** was based on the procedure described by Venkatesh *et al*. [27] The first step was improved by employing microwave irradiation. Thus, the reaction of nitroethene **1** with *p*-nitroaniline using ethanol as a solvent was irradiated at 70 W and 110°C for 90 min, furnishing nitroketene *N,S*-arylaminoacetal **2** in 91% yield.^27^ The cyclization of **2** using POCl_3_ yielded quinoxaline **3**, which was oxidized with *m*-chloroperbenzoic acid to furnish quinoxaline **4** in 87% yield (Fig. 1).

### Activity against Epimastigote Forms

**Figure 2:**
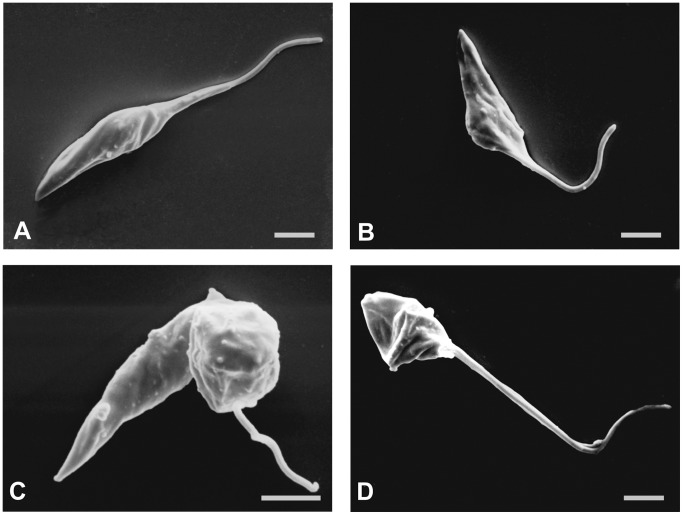
Morphological alterations (SEM) in *T. cruzi* epimastigotes treated with quinoxaline 4. Parasites were treated for 72(a). The epimastigotes treated with the IC_50_ (1.1 µM) presented a wrinkled cell surface (b). The epimastigotes treated with the IC_90_ (3.4 µM) showed distortions in the flagellum with swelling of the membrane (c) and a reduction of the cell body (d). Bars = 2 µm.

**Figure 3:**
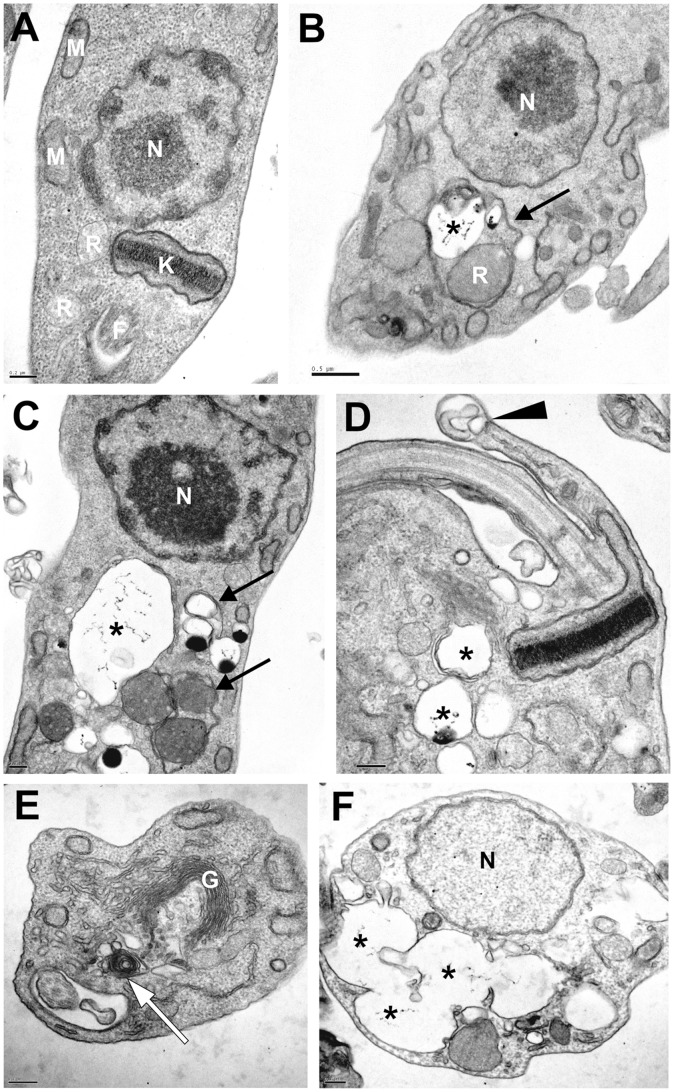
Ultrastructural alterations (TEM) in *T. cruzi* epimastigotes treated with quinoxaline 4. Parasites were treated for 72(A) exhibited organelles with normal morphology. The epimastigotes treated with the IC_50_ (1.1 µM) (B–D) exhibited ER profiles that surrounded organelles (black arrows), autophagosome-like structures (asterisk), and membrane extensions (black arrow head). The parasites treated with the IC_90_ (3.4 µM) (E–F) showed severe alterations in their ultrastructure, exhibiting concentric myelin-like membrane structures (white arrow), distortions in the structure of the Golgi complex, and autophagosome-like structures that comprised a great part of the cytoplasm (F). N, nucleus; R, reservosomes; K, kinetoplast; F, flagellum; M, mitochondrion; G, Golgi complex. Bars = 0.2 µM (A, C–F); Bar = 0.5 µM (B).

Quinoxaline **4** showed effects against all three forms of *T.* cruzi. Against epimastigotes (i.e., the form present in the midgut vector), the drug dose-dependently inhibited parasite growth, with an IC_50_ of 1.1±0.04 µM for 96 h, which was even greater (p≤0.05) than the result found for benznidazole (IC_50_: 8.8±0.04 µM). By monitoring the number of parasites every 24 h, we noticed that a large proportion of the effect was reached after 48 h incubation (Table 1). The same IC_50_ was obtained 1 month later with stock solutions of the drug that were kept in a freezer, showing stability of the drug’s activity (data not shown). The morphological alterations induced by the treatment were assessed by scanning electron microscopy (SEM). The parasites treated with quinoxaline **4** (IC_50_: 1.1 µM; IC_90_: 3.4 µM) showed severe alterations in cell shape, exhibiting a wrinkled cell surface (Fig. 2B), distortion of the flagellum, a reduction of body size (Fig. 2D), and cell membrane swelling (Fig. 2C). The ultrastructural changes induced by quinoxaline treatment for 96 h against epimastigote forms were verified by transmission electron microscopy. The untreated cells presented a normal organelle ultrastructure, such as a prominent nucleus, a ramified mitochondrion, reservosomes, and cellular membranes with preserved structures (Fig. 3A). Treated parasites displayed an altered ultrastructure, showing well-developed profiles of the endoplasmic reticulum (ER) that surrounded organelles, mainly reservosomes (Fig. 3B–C). Diverse autophagosome-like structures could also be seen, such as vacuoles that contained cellular remains (Fig. 3B–D), the formation of myelin-like structures (Fig. 3E), and membranous structures that involved acidocalcisomes (Fig. 3C). These autophagic vacuoles occupied almost all the cytoplasm in epimastigotes treated with the IC_90_ of quinoxaline **4** (Fig. 3F). Parasites treated with quinoxaline **4** also showed an increase in the number of vesicular structures within the cytosol (Fig. 3B–F), intense disorganization of the Golgi complex (Fig. 3E), and the formation of abnormal structures nears the flagellum (Fig. 3D).

**Table 1:** Antiproliferative activity of quinoxaline 4 against epimastigotes of *Trypanosoma cruzi.*

	Quinoxaline 4 (µM)				
Time (h)	0.37	1.84	3.68	18.38	36.76
**24**	1.02±1.77^a^	35.46±8.13	42.85±3.33	64.86±11.86	81.16±18.07
**48**	3.46±5.99	54.39±3.99	72.28±10.86	91.29±0.66	96.20±2.78
**72**	0.76±1.31	53.63±24.26	83.07±12.64	97.72±0.28	100±0.00
**96**	11.38±10.58	63.41±28.07	82.97±19.97	99.82±0.09	100±0.00

^a^ growth inhibition (%) of epimastigotes compared to control. Results presented as median ± standard deviation of three independent experiments.

### Activity against Trypomastigote Forms

**Figure 4:**
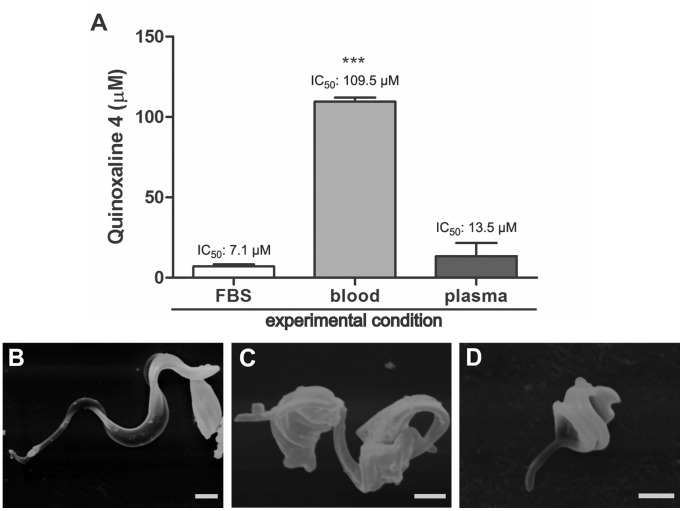
Activity of quinoxaline 4 against trypomastigote forms of *T. cruzi*. (A) Effective concentration (EC_50_) of quinoxaline **4** that inhibited the viability of 50% of the trypomastigotes after 24 h incubation, assays conducted with the addition of FBS, mouse blood or mouse plasma. The experiment was repeated three times. The bars show the median ± standard deviation. *** *p*≤0.01 (B–D) Effects of quinoxaline **4** on the morphology of trypomastigotes. Control parasites (B) exhibited a normal elongated body. The parasites treated with the IC_50_ (C) and IC_90_ (D) showed alterations in regular morphology, expressing shortening and widening of the cell body. Bars = 1 µm.

When quinoxaline **4** was tested for 24 h against trypomastigotes, a dose-dependent inhibition of the viability of the parasites was found, with an EC_50_ of 7.1±1.3 µM. We also assessed the effect of the compound in an experiment with the addition of mouse blood. The results (Fig. 4) showed a striking reduction of the activity of the compound in the presence of mouse blood (EC_50[_: 109.5±2.5 µM). To determine whether this reduction was related to plasma components or the blood cells themselves, the assay was repeated by adding 20% of mouse plasma. No inhibition was observed with the addition of mouse plasma, and the EC_50_ was statistically the same as the one obtained in the regular experiment without blood. To verify morphologic alterations, trypomastigotes were treated with the EC_50_ (7.1 µM) and EC_90_ (15.4 µM) of quinoxaline **4** for 24 h and then analyzed by SEM. The treated parasites showed distortions in cell shape, with shortening and winding of the cell body (Fig. 4).

### Activity against Intracellular Amastigotes

**Figure 5:**
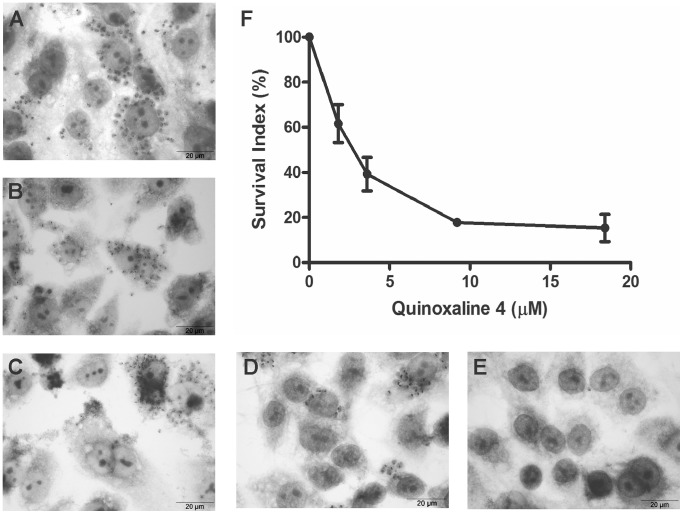
Activity of quinoxaline 4 against intracellular amastigotes of *T. cruzi*. Infected LLCMK_2_ cells were incubated in the presence of different concentrations of quinoxaline **4** for 96 h. Cells were stained with Giemsa, and the number of infected cells and amastigotes inside the cells were counted and used to calculate the survival index (%). (A) Control cells. (B) Quinoxaline **4**, 1.8 µM. (C) Quinoxaline **4**, 3.6 µM. (D) Quinoxaline **4**, 9.2 µM. (E) Quinoxaline **4**, 18.4 µM. (F) Inhibition curve of increasing concentrations of quinoxaline **4** against the survival index (%) of intracellular amastigotes. The experiment was repeated three times. The bars show the median ± standard deviation.

In the present study, quinoxaline **4** also exerted a strong inhibitory effect against intracellular amastigotes. After 96 h incubation in the presence of drug, the number of infected cells and amount of amastigotes inside each cell were reduced compared with the untreated control (Fig. 5). Both parameters are expressed together as the survival index. The compound inhibited the growth of 50% of the amastigotes when the concentration was 2.6±1.2 µM.

### Toxicity Assays

The first experiment that was performed to evaluate the safety of the drug was a cytotoxicity assay in LLCMK_2_ cells. Mammalian cells were exposed to different concentrations of quinoxaline **4**. After 96 h incubation, the concentration that was cytotoxic to 50% of the cells (CC_50_) was 23.3±1.97 µM. By comparing the CC_50_ with the concentration of the drug that inhibited 50% of the parasites, the drug was found to exhibit values of Selective Indices (SI) above one for the clinical relevant forms of *T. cruzi* (SI_trypo_: 3.3; SI_ama_: 8.9), indicating that quinoxaline **4** is more active against the parasite than toxic to mammalian cells *in vitro*. Another way to estimate the toxicity of a compound is to evaluate its hemolytic potential. Red blood cells were incubated in the presence of different concentrations of quinoxaline **4** for 3 h. After incubation, the maximum concentration tested (1000 µM) induced less than 3% hemolysis (data not shown).

### Drug Combination Assay

**Figure 6:**
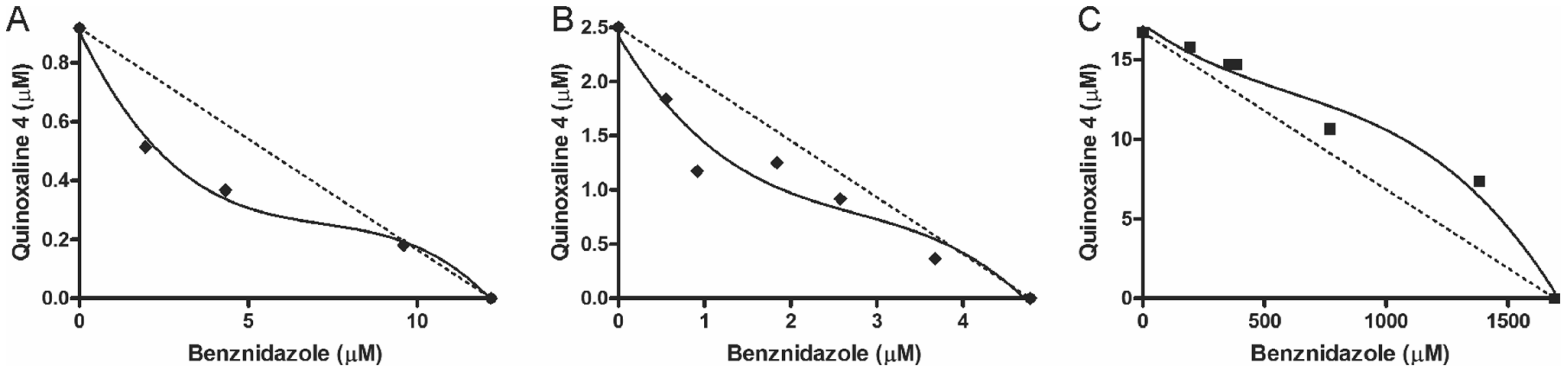
Combination effect of benznidazole and quinoxaline 4. Isobolographs describes the effect of the combination of quinoxaline **4** and benznidazole against epimastigotes (A), trypomastigotes (B), and LLCMK_2_ cells (C). The dotted lines correspond to the additivity effect. Points below the line indicate a synergistic effect. Points above the line indicate an antagonistic effect. The experiment was repeated three times. The points show median values.

The combination of benznidazole and quinoxaline **4** demonstrated promising results (Fig. 6). Strong synergism against epimastigotes was observed. The combination was below the additivity line on the isobolograph, with a combination index (CI) of 0.69. Similar results were obtained in the experiment with trypomastigotes with regard to the profile on the isobolograph and CI of 0.66, confirming the synergistic interaction. Finally, in LLCMK_2_ cells, the drug combination had a CI of 1.04, with all points above the additivity line, indicating an antagonist effect.

### Cell Volume Determination

**Figure 7:**
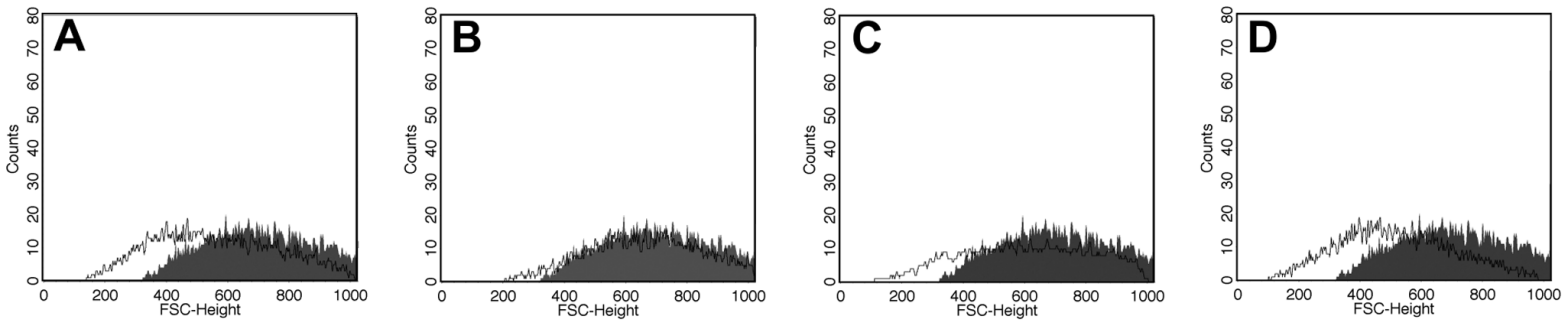
Cell volume alteration of epimastigotes treated with quinoxaline 4. Parasites were treated for 24(counts) and Forward Scatter (FSC) considered as a function of cell size. Gray-filled areas represent untreated cells. Unfilled areas represent treated parasites. (A) Actinomycin D, 20 mM. (B) Quinoxaline **4**, 1.8 µM. (C) Quinoxaline **4**, 3.7 µM. (D) Quinoxaline **4**, 7.4 µM. Typical histograms of at least three independent experiments are shown.

Epimastigote forms treated as previously described were analyzed for Forward Scatter (FSC) by flow cytometry. The histograms in Figure 7 show a dose-dependent reduction of FSC in the cell population treated with quinoxaline **4**. The higher concentration tested (7.4 µM) had an effect similar to actinomycin D treatment (i.e., the positive control).

### Cell Membrane Integrity

**Figure 8:**
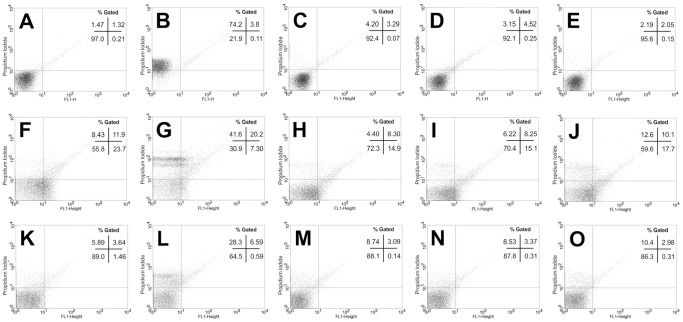
Analysis of cell membrane integrity of parasites treated with quinoxaline 4. Epimastigotes (A–E), amastigotes (F–G), and trypomastigotes were untreated (A, F, K) or treated for 24 h with quinoxaline **4** at 1.8 µM (C, H, M), 3.7 µM (D, I, N), and 7.4 µM (E, J, O) and 40 µM digitonin as a positive control (B, G, L). Events in the upper right and left quadrants represent cells with abnormal membrane permeability. Typical histograms of at least three independent experiments are shown.

Cell membrane integrity in all three parasite forms was unaffected by incubation with quinoxaline **4**. The low number of high fluorescence events (upper left and upper right quadrants) shows cells with abnormal permeability, allowing the entrance of propidium iodide inside the cells. In the three experiments with each parasite form, the values were similar in quinoxaline-treated and untreated parasites (Fig. 8).

## Discussion

Quinoxaline derivatives are a group of heterocyclic compounds characterized by a broad spectrum of biological activity. They are used as therapeutic agents in the treatment of several infections. [13], [16], [17] In the present study, quinoxaline **4** exhibited strong anti-*Trypanosoma cruzi* activity against all of the main forms of the parasite, which was even greater than the activity against epimastigotes determined *in vitro* for benznidazole, the standard drug for treating Chagas’ disease. In a previous study, Estevez [18] synthesized and evaluated the anti-*Trypanosoma* properties of a library of 11 quinoxaline derivatives, all the compounds exhibits trypanocidal activity but none of the tested compounds presented activity higher than quinoxaline **4**.

Another important consideration is the *in vitro* safety of the drug revealed by cytotoxicity and hemolysis tests. Comparing the toxicity against LLCMK_2_ cells with the trypanocidal activity of quinoxaline **4**, it is possible to observe that the drug is clearly selective against all three parasite forms, with higher SIs for the proliferative forms (epimastigote and amastigote). Hemolytic activity is another problem that some toxic drugs present. Amphotericin B is a commercial drug used against fungi and some protozoa, but it is also known to have hemolytic activity, inducing hemolysis in 50% of red blood cells at 50.8 µM. [36] Quinoxaline **4** did not present significant hemolysis at the highest concentration tested (1000 µM).

When the drug was tested in the presence of blood, a substantial reduction of activity against trypomastigotes was found. Both a decrease and complete inhibition of the activity of a trypanocidal compound with the addition of blood were observed previously, explained by an interaction between the drug and serum proteins or oxyhaemoglobin. [37]–[38] To verify whether this inhibition was related to an interaction with serum or the blood cells themselves, the same experiment was conducted using mouse plasma. In the presence of mouse serum, the same EC_50_ as the one found in the regular experiment with FBS was found, indicating that the drug may interact with blood cells through incorporation into red blood cells or linking to surface proteins, [39] such that the drug was unable to act against the free trypomastigotes during the 24 h incubation. The scenario for this interaction *in vivo* may be different when the exposure time is longer than in *in vitro* experiments and the storage and transport of the drug by erythrocytes may extend the half-life and decrease the toxicity profile. [40].

A promising interaction between quinoxaline **4** and benznidazole was also found. The combination was synergistic against epimastigotes and trypomastigotes and antagonistic against LLCMK_2_ cells. Several studies demonstrated the potential of the combination of benznidazole with various drugs against *T. cruzi*. [12], [41] Based on these results, one question that arises is the mechanism by which the drug combination is synergistic. According to Chou, [42] predicting the mechanism of action of two drugs based on their synergistic interaction is difficult. The mechanism of such a combination can be one specific point of action or innumerable steps. However, several studies have demonstrated that compounds with different mechanisms of action can cooperate to enhance the final effect observed. [43]–[45] Combination therapy with new drugs has been shown to be an important approach against *T. cruzi*. The combination of different enzymatic inhibitors of the ergosterol biosynthesis pathway, a specific target for fungi and protozoa, can promote the cure of infected mice at low concentrations of both drugs. [44] Amiodarone, an ergosterol biosynthesis blocker, exerted an intrinsic synergistic effect when combined with Posaconazole against *T. cruzi.* [45] Another study performed with Chagas’ disease patients found that combinatory therapy with benznidazole and allopurinol was promising, reflected by a reduction of parasite burden. [25].

The combination of benznidazole, nifurtimox, and posaconazole was found to be effective in the complete cure of mice that were chronically or acutely infected by the Tulahuen strain of *T. cruzi*, but the same effect was not observed for the Y strain. This synergy was explained by the authors as cooperation between the mechanisms of action of the drugs. [46] Benznidazole and nifurtimox are prodrugs that induce oxidative stress and generate electrophilic metabolites in the parasite through different pathways. [6] Posaconazole acts by inhibiting lanosterol demethylase in the parasite, preventing the *de novo* synthesis of ergosterol. [47] Thus, the effect of the combination of quinoxaline **4** and benznidazole may also involve different cooperative mechanisms of action.

The mechanism of action of benznidazole is well established. The nitro group of the drug is reduced by nitroreductases of the protozoan, with the formation of various free radical intermediates that induce oxidative stress and electrophilic metabolites. These metabolites are responsible for the main mechanism of action of benznidazole. They covalently bond to macromolecules, such as DNA, proteins, and lipids, and induce the death of parasite. [6], [47] The mechanism of action of quinoxaline **4**, in contrast, has not been elucidated. A previous docking study with different quinoxaline derivatives demonstrated a strong interaction with the poly (ADP-ribose) polymerase of *T. cruzi*, [18] an enzyme implicated in the DNA-damage response and signaling of different cell death pathways. [48] Several quinoxaline derivatives are also known to have strong anti-tumor activity. XK469 (2-{4-[(7-chloro-2-quinoxalinyl)oxy]phenoxy}propionic acid) and SH80 ([2-{4-[(7-bromo-2-quinolinyl)oxy]phenoxy}]propionic acid) are two chemotherapeutic agents that are effective against a broad range of human tumors and related to the induction of cell cycle arrest and a mixed autophagy and apoptosis profile. [49] In the present study, the treatment with quinoxaline **4** led to a reduction of the cell volume of the epimastigotes and did not induce severe alterations in the cell membrane integrity of the three parasite forms at the maximum concentration tested (7.4 µM). This was also confirmed by the TEM analysis of the epimastigotes, showing that the majority of the cells had a well-preserved external membrane, even when the inside of the cell was completely altered. The SEM analysis showed almost no cells with leakage of cellular content. Altogether, these results exclude the possibility that death occurred through classic necrosis, which is well characterized by an increase in cell volume, swelling of the organelles, plasma membrane rupture, and subsequent loss of intracellular content. [50]–[51].

The ultrastructure of quinoxaline-treated epimastigotes also revealed signs of the classic programmed cell death type II (PCD II; i.e., autophagic cell death), which is morphologically characterized by autophagosome formation and the appearance of membranes that surround organelles and concentric cytosolic membrane structures. [36], [52], [53] The well-developed ER profiles that surround organelles, especially reservosomes, observed in quinoxaline-treated epimastigotes was also found in *T. cruzi* after treatment with a triazolic naphthoquinone. [54] Another alteration found was secretory pathway stress, expressed as numerous vesicles in the cytoplasm with severe disturbance of the ultrastructure of the Golgi complex, which may indicate that this organelle was acting as a source of the membranes for the expansion of phagophores, a process already verified in yeast autophagosome formation. [55] In summary, our results strongly indicate parasite PCD through an autophagic pathway, which is promising for possible future therapeutic approaches because this is a controlled process of death characterized by a lack of tissue inflammatory response in the host. [56].

## Conclusion

The quinoxaline derivative tested in the present study represents a possible scaffold in the complex process of development of a new drug against *T. cruzi*. The results achieved in the drug combination assay with quinoxaline **4** and benznidazole are also promising. The syntheses of new derivates could result in enhanced molecules with still higher selective indices. Further studies are necessary to elucidate how exactly autophagic cell death is involved in the mechanism of action of quinoxaline **4** and how both drugs can interact synergistically and result in an enhanced final effect.
